# A randomised controlled trial of dietary improvement for adults with major depression (the ‘SMILES’ trial)

**DOI:** 10.1186/s12916-017-0791-y

**Published:** 2017-01-30

**Authors:** Felice N. Jacka, Adrienne O’Neil, Rachelle Opie, Catherine Itsiopoulos, Sue Cotton, Mohammedreza Mohebbi, David Castle, Sarah Dash, Cathrine Mihalopoulos, Mary Lou Chatterton, Laima Brazionis, Olivia M. Dean, Allison M. Hodge, Michael Berk

**Affiliations:** 1 IMPACT Strategic Research Centre, Deakin University, Geelong, VIC Australia; 2 School of Population Health, The University of Melbourne, Melbourne, VIC Australia; 3 Orygen, The National Centre of Excellence in Youth Mental Health, Parkville, VIC Australia; 4 Department of Psychiatry, University of Melbourne, Melbourne, VIC Australia; 5 School of Allied Health, La Trobe University, Melbourne, VIC Australia; 6 Department of Medicine, The University of Melbourne, Melbourne, VIC Australia; 7 Centre for Population Health Research, Deakin University, Geelong, VIC Australia; 8 Cancer Epidemiology and Intelligence Division, Cancer Council Victoria, Carlton, VIC Australia; 9 Centre for Adolescent Health, Murdoch Childrens Research Institute, Melbourne, VIC Australia; 10 Black Dog Institute, Randwick, NSW Australia; 11 St Vincents Hospital, Fitzroy, VIC Australia; 12 The Florey Institute of Neuroscience and Mental Health, Parkville, VIC Australia; 13 Food & Mood Centre, Deakin University, IMPACT SRC, School of Medicine, PO Box 281, Geelong, 3220 Victoria Australia

**Keywords:** Depression, Major depressive disorder, Diet, Nutrition, Randomised controlled trial, Dietetics

## Abstract

**Background:**

The possible therapeutic impact of dietary changes on existing mental illness is largely unknown. Using a randomised controlled trial design, we aimed to investigate the efficacy of a dietary improvement program for the treatment of major depressive episodes.

**Methods:**

‘SMILES’ was a 12-week, parallel-group, single blind, randomised controlled trial of an adjunctive dietary intervention in the treatment of moderate to severe depression. The intervention consisted of seven individual nutritional consulting sessions delivered by a clinical dietician. The control condition comprised a social support protocol to the same visit schedule and length. Depression symptomatology was the primary endpoint, assessed using the Montgomery–Åsberg Depression Rating Scale (MADRS) at 12 weeks. Secondary outcomes included remission and change of symptoms, mood and anxiety. Analyses utilised a likelihood-based mixed-effects model repeated measures (MMRM) approach. The robustness of estimates was investigated through sensitivity analyses.

**Results:**

We assessed 166 individuals for eligibility, of whom 67 were enrolled (diet intervention, *n* = 33; control, *n* = 34). Of these, 55 were utilising some form of therapy: 21 were using psychotherapy and pharmacotherapy combined; 9 were using exclusively psychotherapy; and 25 were using only pharmacotherapy. There were 31 in the diet support group and 25 in the social support control group who had complete data at 12 weeks. The dietary support group demonstrated significantly greater improvement between baseline and 12 weeks on the MADRS than the social support control group, *t*(60.7) *=* 4.38, *p <* 0.001, Cohen’s *d* = –1.16. Remission, defined as a MADRS score <10, was achieved for 32.3% (*n* = 10) and 8.0% (*n* = 2) of the intervention and control groups, respectively (*χ* ^*2*^ (1) = 4.84, *p* = 0.028); number needed to treat (NNT) based on remission scores was 4.1 (95% CI of NNT 2.3–27.8). A sensitivity analysis, testing departures from the missing at random (MAR) assumption for dropouts, indicated that the impact of the intervention was robust to violations of MAR assumptions.

**Conclusions:**

These results indicate that dietary improvement may provide an efficacious and accessible treatment strategy for the management of this highly prevalent mental disorder, the benefits of which could extend to the management of common co-morbidities.

**Trial registration:**

Australia and New Zealand Clinical Trials Register (ANZCTR): ACTRN12612000251820. Registered on 29 February 2012.

## Background

There is now extensive observational evidence across countries and age groups supporting the contention that diet quality is a possible risk or protective factor for depression [1–5]. Although there are many versions of a ‘healthful diet’ in different countries and cultures, the available evidence from observational studies suggests that diets higher in plant foods, such as vegetables, fruits, legumes and whole grains, and lean proteins, including fish, are associated with a reduced risk for depression, whilst dietary patterns that include more processed food and sugary products are associated with an increased risk of depression [1, 6, 7]. Whilst cognisant of the limitations of observational data, these associations are usually observed to be independent of socioeconomic status, education and other potentially confounding variables and not necessarily explained by reverse causality (see, e.g. [7–10]).

Recently, a meta-analysis confirmed that adherence to a ‘healthful’ dietary pattern, comprising higher intakes of fruit and vegetables, fish and whole grains, was associated with a reduced likelihood of depression in adults [1]. Similarly, another meta-analysis reported that higher adherence to a Mediterranean diet was associated with a 30% reduced risk for depression, with no evidence for publication bias [11]. The Mediterranean diet is recognised as a healthful dietary pattern and has been extensively associated with chronic disease risk reduction [12]. More recently, a systematic review confirmed relationships between unhealthful dietary patterns, characterised by higher intakes of foods with saturated fat and refined carbohydrates, and processed food products, and poorer mental health in children and adolescents [2]. Several cohort studies also reported associations between the quality of women’s diets during pregnancy and the risk for emotional dysregulation in children [13–15], with new insights into potential mechanisms of action that include brain plasticity [16], the gut microbiota [17] and inflammatory [18] and oxidative stress [19] pathways.

Although there are data suggesting that some nutritional supplements may be of utility as adjunctive therapies in psychiatric disorders [20], the field of research focusing on the relationships between overall dietary quality and mental disorders is new and has thus far been largely limited to animal studies and observational studies in humans. Thus, whilst the existing observational data support a causal relationship between diet quality and depression on the basis of the Bradford Hill criteria [3] and are supported by extensive experimental data in animals (see, e.g. [21]), randomised controlled trials are required to test causal relationships and identify whether or not dietary change can improve mental health in people with such conditions. We conducted a systematic review and identified a number of interventions with a dietary change component that had examined mental health-related outcomes [22]. Whilst approximately half of these studies reported improvements in measures of depression or anxiety following the intervention, at the time of the review no studies fulfilling quality criteria had been conducted in mental health populations or had been designed to test the hypothesis that dietary improvement might result in improvements in mental health. Since then, one study has been published evaluating the possible impact of a lifestyle program, comprising both diet and exercise, on mental health symptoms in patients with depression and/or anxiety; this study failed to show any differences in symptom levels between those in the intervention and those in the attention control group [23]. On the other hand, post hoc analysis of a large-scale intervention trial provides preliminary support for dietary improvement as a strategy for the primary prevention of depression. Individuals at increased risk for cardiovascular events were randomised to a Mediterranean diet supplemented with either extra-virgin olive oil or mixed nuts, or a low-fat control diet [12]. Whilst not statistically powered to assess the effectiveness of the intervention for preventing depression, there was evidence (albeit non-significant) of a reduced risk for incident depression for those randomised to a Mediterranean diet with nuts. This protective effect was statistically significant in those with type 2 diabetes, who comprised approximately half the sample [24].

Using a randomised controlled trial (RCT) design, we thus aimed to investigate the efficacy of a dietary program for the treatment of major depressive episodes. In this trial, Supporting the Modification of lifestyle In Lowered Emotional States (SMILES), we hypothesised that structured dietary support, focusing on improving diet quality using a modified Mediterranean diet model, would be superior to a social support control condition (befriending) in reducing the severity of depressive symptomatology.

## Methods

### Study design

This was a 12-week, parallel-group, single blind RCT of a dietary intervention in the treatment of moderate to severe depression (for the protocol see [25]). This trial was registered in the Australia and New Zealand Clinical Trials Register (ANZCTR): (ACTRN12612000251820) prior to commencing recruitment. Participants were recruited from two sites: Barwon Health in Geelong and St. Vincent’s Health in Melbourne (Victoria, Australia) over a 3-year period. Participants were randomised to receive either dietary support or social support (‘befriending’ [26]). Participants in both groups completed assessments prior to program commencement (baseline), with the primary and secondary outcomes measured at program completion (12 weeks, primary endpoint). Approval to conduct the study was received from Human Research Ethics Committees of St. Vincent’s and Barwon Health. Written informed consent was obtained from all participants after they had received a complete description of the study. The study’s protocol was developed in accordance with the Standard Protocol Items: Recommendations for Interventional Trials (SPIRIT) guidelines. Reporting of findings pertaining to primary and secondary outcomes was done in accordance with the Consolidated Standards of Reporting Trials (CONSORT) 2010 guidelines and their extension to non-pharmacologic treatments.

### Participants

#### Inclusion criteria

Eligibility criteria included participants who were at screening: aged 18 or over and could provide informed consent; successfully fulfilled the Diagnostic and Statistical Manual of Mental Disorders (4th ed.; DSM-IV-TR) diagnostic criteria for a major depressive episode (MDE); scored 18 or over on the Montgomery–Åsberg Depression Rating Scale (MADRS) [27]; and scored 75 or less, out of a possible score of 104, on a Dietary Screening Tool (DST) [28] modified for Australian food products. The DST was completed to confirm ‘poor’ dietary quality, before enrolment. This screening tool was used to reflect usual daily or weekly intake of specified foods. Broadly defined, participants had to report a poor (low) intake of dietary fibre, lean proteins and fruit and vegetables, and a high intake of sweets, processed meats and salty snacks. If participants were on antidepressant therapy or undergoing psychotherapy, they were required to be on the same treatment for at least 2 weeks prior to randomization. Participants had to be readily available for a 12-week period and have the ability to eat foods as prescribed, without religious, medical, socio-cultural or political factors precluding participation or adherence to the diet.

#### Exclusion criteria

Participants were ineligible if they had: (1) a concurrent diagnosis of bipolar I or II disorder; (2) two or more failed trials of antidepressant therapy for the current MDE; (3) known or suspected clinically unstable systemic medical disorder; (4) pregnancy; (5) commencement of new psychotherapy or pharmacotherapy within the preceding 2 weeks; (6) severe food allergies, intolerances or aversions; (7) current participation in an intervention targeting diet or exercise; (8) a primary clinical diagnosis of a personality disorder and/or a current substance use disorder.

### Sample recruitment

Community-based recruitment strategies were used to identify study participants, including flyers in medical waiting rooms, pharmacies and university campuses; newsletters; and contact with potential referral sources (e.g. general practitioners, private psychiatrists and local psychiatric inpatient units). Media interviews and advertisements in social media (e.g. Twitter, Facebook), Google, local newspapers and radio stations were also employed as recruitment strategies. Ethics committee requirements meant that we needed to be explicit regarding our planned intervention, with the advertisements stating: ‘We are trialling the effect of an educational and counselling program focusing on diet that may help improve the symptoms of depression’.

### Interventions

#### Dietary support

The dietary intervention comprised personalised dietary advice and nutritional counselling support, including motivational interviewing, goal setting and mindful eating, from a clinical dietician in order to support optimal adherence to the recommended diet. This comprised the ‘Mod*i*MedDiet’, developed by RO and CI, which was based on the Australian Dietary guidelines [29] and the Dietary Guidelines for Adults in Greece [30] and is concordant with our previous dietary recommendations for the prevention of depression [31]. The primary focus was on increasing diet quality by supporting the consumption of the following 12 key food groups (recommended servings in brackets): whole grains (5–8 servings per day); vegetables (6 per day); fruit (3 per day), legumes (3–4 per week); low-fat and unsweetened dairy foods (2–3 per day); raw and unsalted nuts (1 per day); fish (at least 2 per week); lean red meats (3–4 per week) [32], chicken (2–3 per week); eggs (up to 6 per week); and olive oil (3 tablespoons per day), whilst reducing intake of ‘extras’ foods, such as sweets, refined cereals, fried food, fast-food, processed meats and sugary drinks (no more than 3 per week). Red or white wine consumption beyond 2 standard drinks per day and all other alcohol (e.g. spirits, beer) were included within the ‘extras’ food group. Individuals were advised to select red wine preferably and only drink with meals. The dietary composition of the Mod*i*MedDiet was as follows: protein 18% of total energy (E); fat 40% of E; carbohydrates 37% of E; alcohol 2% of E; fibre/other 3% of E. The diet was designed to be easy to follow, sustainable, palatable, and satiating. Individuals were advised to consume the diet *ad libitum*, as the intervention did not have a weight loss focus. The method for scoring the Mod*i*MedDiet is similar to those used in PREDIMED [33] and the Framingham Offspring Cohort [34]. It is a criterion-based diet score that uses pre-defined absolute or normative goals of consumption for specific food items, independent of the individual’s characteristics. It was developed based on the recommended intakes of the 11 food group components that comprise the Mod*i*MedDiet (as above), and of the score has a theoretical maximum value of 120.

Participants received seven individual dietary support sessions of approximately 60 minutes each, delivered by an Accredited Practising Dietician; the first four sessions occurred weekly and the remaining three sessions occurred every 2 weeks. At the first session, the dietician conducted a diet history to assess usual dietary intake. Participants were provided with supporting written information specifically designed for the intervention to assist with achieving dietary adherence. In order to provide examples of serving sizes and exposure to the recommended foods, participants were also provided with a food hamper, incorporating the main components of the diet, along with recipes and meal plans. Subsequent sessions used motivational interviewing techniques, and participants were encouraged to set personalised goals.

#### Social support

The social support control condition comprised a manualised ‘befriending’ protocol [26], using the same visit schedule and length as the dietary support intervention. Befriending consists of trained personnel discussing neutral topics of interest to the participant, such as sport, news or music, or in cases where participants found the conversation difficult, engaging in alternate activities such as cards or board games, with the intention of keeping the participant engaged and positive. This is done without engaging in techniques specifically used in the major models of psychotherapy. Research assistants (RAs) in this trial completed manual-guided training and also participated in role-playing training exercises to ensure consistent delivery of the protocol. Befriending aims to control for four factors: time; client expectancy; therapeutic alliance; and therapist factors when compared to the intervention group in an RCT and is often used as a control condition for clinical trials of psychotherapy [26]. Participants in the social support control group were provided with movie tickets as compensation for their time and participation in the study and were offered participation in a group dietary counselling session at the conclusion of the trial.

### Assessments and outcomes

Once deemed to be eligible, participants completed a 7-day food diary and the Cancer Council of Victoria food frequency questionnaire [35], in the week leading up to baseline assessment. Participants attended a local pathology clinic to provide fasting blood samples before undertaking baseline assessment and randomization.

#### Baseline and follow-up assessments

Details of baseline and follow-up assessments have been reported elsewhere [25]. Briefly, primary and secondary endpoints were as described in the following sections.

##### Primary outcome

The MADRS was used to assess the change in depressive symptomatology at baseline and at the primary endpoint of 12 weeks. The MADRS is an interviewer-rated instrument, comprising 10 items, each measured on a 6-point scale (scores range from 0–60 with higher scores depicting greater symptom severity). It has been found to be a robust and psychometrically sound measure of depressive symptomatology [27].

##### Secondary outcomes

The Hospital Anxiety and Depression Scale (HADS) [36] was administered as a self-report questionnaire. The Profile of Mood States (POMS) was used to assess mood [37], and the Clinical Global Impression - Improvement (CGI-I) Scale [38] was used to assess change in symptoms from baseline to endpoint. The World Health Organization wellbeing scale (WHO-5) [39] and the Generalized Self-Efficacy Scale [40] were used to assess wellbeing and self-efficacy, respectively. Clinical data including height, weight and waist circumference were also collected and the body mass index (BMI) was calculated. Participants were also asked the following: whether they were a current smoker (yes/no); if they had an existing medical condition (physical or mental); and the names and doses of any medications they were taking. Current levels of physical activity were assessed using International Physical Activity Questionnaire (IPAQ) scores, which capture Metabolic Equivalent of Task (MET) minutes per week. A total MET score was calculated for each participant as a summary of Walking, Moderate and Vigorous MET scores [41]. Dietary quality was assessed using the Mod*i*MedDiet score, which was based on consumption of the key food groups (i.e. wholegrains, vegetables, fruits, legumes, nuts, fish, lean red meats, chicken, low fat dairy, eggs, olive oil, extras) and will be presented in more detail, along with the dietary strategy, in a forthcoming publication. Dietary assessments, using 7-day food diaries, were administered at baseline and endpoint to both groups to identify dietary changes and adherence to the recommended diet; this was done by assessing change in the Mod*i*MedDiet score, which is based on the consumption of the key food groups. Biomarkers, including plasma fatty acids, fasting glucose, total and HDL and LDL cholesterol and triglycerides were also assessed.

#### Sample size

Our original sample size calculation required 88 people per group, assuming an attrition of 15%, with 8 predictors. For a one-tailed analysis with type I error or alpha set at the .05 level, the study would have been powered at 80% to detect a true difference in rating scale score between the diet and befriending groups if the effect size was 0.15 or greater on the MADRS.

#### Randomization

The randomization sequence was computer generated by an independent person (OD) using a 2 × 2 block design. The sequence was saved to a password-protected spreadsheet, and groups were coded A and B. The randomization allocation was managed by the trial dieticians or ‘befrienders’, in order to ensure that the research assistants responsible for mental health assessments were blind to participants’ group allocations, and the randomization schedule and coding of group allocations were not, at any time, accessible to the research assistants conducting the assessments, or to the biostatistician (SC). At the conclusion of the baseline appointment, the dietician/befriender would meet privately with the participant and inform them of their group allocation in order to maintain blinding of the research assistants.

#### Blinding

Although full blinding of participants to condition in this study was not possible, several strategies were employed to reduce the risk of bias. First, participants were provided with only partial information on the study hypothesis; the social support control condition was termed ‘befriending’ and research assistants emphasised the link between social support and mental health as an outcome of interest; and participants in both the intervention and the social support control group were provided with standardised care, with all participants attending appointments in the same location and with the same format, as well as similar duration and frequency. All communication between participants and research staff during the period of intervention (i.e. scheduling concerns, questions regarding intervention) was done directly between participants and their respective ‘clinician’. Participants were clearly instructed only to contact the dietician/befriender personally and to avoid contact with the research assistant, and voice messages were checked daily by the dietician/befriender to avoid unintended contact or information on participants’ allocation. Research assistants did not have direct contact with participants for the duration of the intervention. Final assessments were organised by the dietician or befriender, and research assistants remained blind to condition for the final assessment of outcomes. Prior to assessment, participants were reminded not to reveal the group to which they had been assigned. Statistical analyses were conducted by an external statistician (SC), who was blind to group allocation prior to analysis.

#### Data analyses

The analyses were conducted in accordance with the International Conference on Harmonization E9 statistical principles. Independent samples *t* tests and chi-square (*χ* ^*2*^) analyses were used to compare participants who completed and did not complete the 12 weeks of the trial.

Intention-to-treat (ITT) analyses were adopted. The primary efficacy analysis was based on between-group differences in average change from baseline to 12 weeks for the primary outcome measure (MADRS); these analyses were conducted using planned comparisons within a restricted maximum likelihood (REML)-based mixed-effects model, repeated measures (MMRM) approach. Within the MMRM, treatment and assessment occasion and the interaction between treatment group and assessment occasion were included as fixed factors. The MMRM approach is the preferred method of dealing with clinical trial data in psychiatry [42]. The benefits of these MMRM methods are that all available participant data are included in the model [42]. By planning to use MMRM, we made the a priori assumption that missing data were missing at random (MAR); however, we tested these assumptions in sensitivity analyses (as below). The Toeplitiz covariance structure was used to model the relations between observations on different occasions. Planned comparisons using MMRM were also conducted to examine group differences in mean change on the secondary outcome measures from baseline to 12 weeks. Cohen’s *d* as a measure of effect size was calculated based on observed data. Supplementary sensitivity analyses with the MMRM models were conducted, controlling for relevant confounding variables such as gender, education, physical activity, baseline BMI and baseline Mod*i*MedDiet score. All tests of treatment effects were conducted using an alpha level of 0.05 and reporting 95% confidence intervals. Pearson’s product-moment correlations were calculated to determine whether changes in MADRS scores correlated to changes in biomarkers. Analysis of covariance (ANCOVA) was implemented to evaluate interactions between group allocation and change adherence to Mod*i*MedDiet on MADRS scores at 12 weeks, adjusting for MADRS at baseline. Whilst acknowledging the increased potential for type 1 errors, given that reported comparisons for all primary and secondary outcomes were pre-planned comparisons that were determined a priori and documented in the trial protocol, we did not make adjustments for multiple comparisons.

#### Sensitivity analyses

We compared demographic, health measures, current treatment, diet quality and psychological measures at baseline between participants with complete follow-up and those with missing data at follow-up, using the chi-squared test for categorical data and *t* tests for continuous measures. To test departures from missing at random (MAR), a weighted sensitivity analysis using the Selection Model Approach was applied to the main outcome findings [43, 44]. Briefly, once data had been imputed under MAR (*n* = 5), parameter estimates from each imputed dataset were reweighted to allow for the data to be missing not at random (MNAR). The chosen constant values used to add to the imputed missing data to account for MNAR were multiplications of standard error (i.e. 1.6) for main outcome comparison under MAR assumptions. To evaluate the robustness of our findings, different degrees of departure from the MAR assuming plausible values ranging from 10*SE to –8*SE were considered.

## Results

**Fig. 1:**
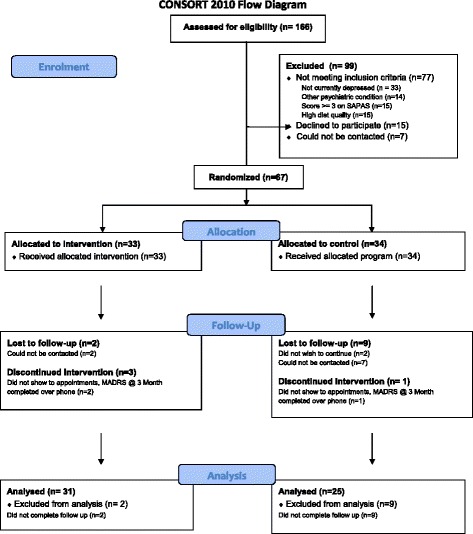
CONSORT flow chart

We assessed 166 individuals for eligibility. Of these, 99 were excluded. We thus randomised 67 individuals with MDD to the trial (intervention, *n* = 33; social support control, *n* = 34). Figure 1 presents a CONSORT flow chart. Baseline characteristics of all enrolled participants are presented in Table 1. The dietary group had significantly lower scores on the dietary screening tool and the Mod*i*MedDiet score than the social support control group at baseline, primarily due to lower intakes of fruit and higher intakes of *extras*. Otherwise, groups were well matched on characteristics.

**Table 1:** Baseline characteristics of all those randomised to the dietary support (DS) and social support (SS) groups

		Total (*n* = 67)	DS (*n* = 33)	SS (*n* = 34)
Demographic				
Gender % female	*% (n)*	71.6 (48)	81.8 (27)	61.8 (21)
Age	*M (SD)*	40.3 (13.1)	37.5 (10.7)	43.1 (14.6)
Post-secondary school education	*% (n)*	51.5 (34)	51.5 (17)	51.5 (17)
Household income				
Above $80,000 per annum	*% (n)*	23.1 (15)	25.0 (8)	21.2 (7)
Health measures				
BMI	*M (SD)*	29.5 (8.0)	30.0 (9.3)	29.0 (6.5)
Current smoker	*% (n)*	14.1 (9)	10.0 (3)	17.6 (6)
Comorbid disorder	*% (n)*	71.2 (47)	75.0 (24)	67.6 (23)
Number of comorbid disorders	*M (SD)*	1.5 (1.4)	1.6 (1.4)	1.5 (1.4)
Physical activity (IPAQ score)	*M (SD)*	2336 (2585)	2146 (2565)	2509 (2629)
Current treatments				
Psychopharmacotherapy	*% (n)*	68.7 (46)	75.8 (25)	61.8 (21)
Psychological therapy	*% (n)*	44.8 (30)	39.4 (13)	50.0 (17)
Diet quality				
Screen of diet quality	*M (SD)*	51.2 (11.0)	48.5 (10.3)	53.9 (11.3)
Mod*i*MedDiet (0-120)	*M (SD)*	41.5 (14.3)	36.2 (12.8)	47.3 (13.7)
Psychological measures				
MADRS (0-60)	*M (SD)*	25.4 (4.6)	26.1 (4.9)	24.7 (4.2)
HADS – (D) (0-21)	*M (SD)*	9.6 (3.7)	10.0 (4.0)	9.2 (3.4)
HADS – (A) (0-21)	*M (SD)*	11.7 (3.0)	12.1 (3.1)	11.2 (2.8)

*BMI* body mass index, *MADRS* Montgomery-Åsberg Depression Rating Scale, *HADS* Hospital Anxiety and Depression Scale

### Completer analysis

Fifty-six individuals (83.6%) completed the assessment at the 12-week endpoint. There were significantly more completers in the dietary support group (93.9%, *n* = 31) than the social support control group (73.5%, *n* = 25), *χ* ^*2*^ (1) = 5.08, *p* = 0.024. Those who did not complete the intervention were significantly more likely to have post-secondary education (81.8%, *n* = 9) than those who completed (45.5%, *n* = 25), *χ* ^*2*^ (1) = 4.85, *p* = 0.028; this relationship was observed for the social support control group, *χ* ^*2*^ (1) = 6.92, *p* = 0.009 and not in the dietary support group, *χ* ^*2*^ (1) = 0.01, *p* = 0.965.

#### Primary outcome: depressive symptomatology

**Fig. 2:**
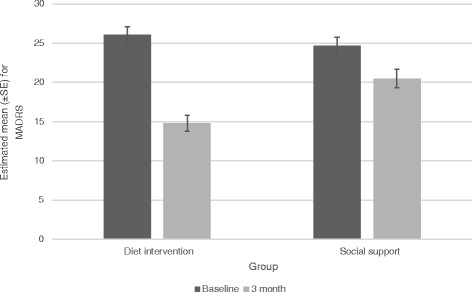
MADRS scores for dietary support and social support control groups at baseline and endpoint. Effect size: Cohen’s *d* = –1.16 (95% CI –1.73, –0.59). Baseline data *n* = 67; 12 week data *n* = 56

The dietary support group demonstrated significantly greater improvement in MADRS scores between baseline and 12 weeks than the social support control group, *t*(60.7) *=* 4.38, *p <* .001 (Fig. 2). The effect size for this difference was a Cohen’s *d* of –1.16 (95% CI –1.73, –0.59) and represented an estimated average between group difference, in terms of change from baseline to 12 weeks, of 7.1 points on the MADRS (*SE* = 1.6). The MMRM was rerun, adjusting for variables such as sex, education, physical activity, baseline BMI and baseline Mod*i*MedDiet score; the significant between-group difference in change from baseline to 12 weeks remained, *t*(58.7) *=* 4.40, *p <* 0.001.

**Fig. 3:**
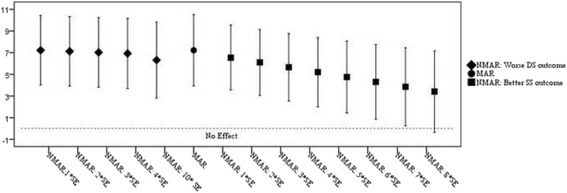
Weighted sensitivity analyses using the Selection Model Approach for MADRS scores, accounting for missing data under the non-missing at random (*NMAR*) assumption

Results from sensitivity analyses accounting for missing data under the NMAR assumption are presented in Fig. 3. Two NMAR scenarios were investigated in the sensitivity analyses: (1) dropouts in the intervention group had worse MADRS outcome at 12 weeks, and (2) dropouts in the control group had better MADRS outcomes. As Fig. 3 shows, findings were insensitive to assumption 1, even when assuming outcomes as large as 10*SE (an increase of 16 in MADRS score compared to imputation under the MAR assumption). Findings were also robust under assumption 2, and only a large departure from the MAR assumption (i.e. 8*SE = 12.8 reduction on MADRS) made the observed intervention effect non-significant.

#### Secondary outcomes

At 12 weeks, 32.3% (*n* = 10) of the dietary support group and 8.0% (*n* = 2) of the social support control group achieved remission criteria of a score less than 10 on the MADRS; this between-group difference was significant, *χ* ^*2*^ (1) = 4.84, *p* = 0.028. Based on these remission data, the number needed to treat (NNT) is 4.1 (95% CI of NNT 2.3–27.8).

Concordant with the findings for the MADRS, the dietary support group demonstrated significantly greater improvement from baseline to 12 weeks than the social support control group on the Hospital Anxiety and Depression Scale (HADS)-depression subscale, *t*(55.1) = 2.20, *p* = 0.032 (Table 2). Similar findings were obtained with the HADS-anxiety subscale, *t*(59.0) = 2.19, *p* = 0.033. These significant differences remained after controlling for sex, education, physical activity, baseline BMI and baseline Mod*i*MedDiet scores. Cohen’s *d* for HADS-depression was –0.632 (95% CI –1.186, –0.078), and for HADS-anxiety it was –0.594 (95% CI –1.147, –0.042).

**Table 2:** Mean (±standard error) estimates derived from mixed model repeated measures (MMRM, unadjusted estimates) comparing differences between the dietary support (DS) and social support (SS) groups in terms of changes from baseline to primary endpoint of 12 weeks

Characteristic	DS		SS		Between-group differences in change from baseline to 12 weeks				
	Baseline	12 weeks	Baseline	12 weeks					
	M (SE)	M (SE)	M (SE)	M (SE)	M (SE)	95% CI [LCI, UCI]	*t* ^a^	df	*p*
Symptoms									
MADRS (0-60)	26.1 (1.0)	14.8 (1.1)	24.7 (1.0)	20.5 (1.2)	7.1 (1.6)	3.9, 10.4	4.38	60.7	<.001
HADS – (D) (0-21)	10.0 (0.6)	5.3 (0.7)	9.2 (0.6)	6.8 (0.7)	2.3 (1.1)	0.2, 4.4	2.20	55.1	.032
HADS – (A) (0-21)	12.1 (0.6)	8.4 (0.6)	11.2 (0.6)	9.5 (0.7)	2.0 (0.9)	0.2, 3.9	2.19	59.0	.033
Mood									
POMS (-32–200)	54.5 (6.0)	30.3 (6.5)	40.9 (5.8)	32.1 (6.7)	15.4 (9.9)	-4.6, 35.3	1.56	43.5	.127
Self-efficacy									
GSE (10-40)	24.6 (1.0)	28.1 (1.0)	25.7 (1.0)	26.7 (1.1)	-2.4 (1.6)	-5.7, 0.9	-1.44	59.0	.156
Wellbeing									
WHO-5 (0-25)	6.9 (0.8)	12.3 (0.9)	6.7 (0.8)	10.3 (1.0)	-1.8 (1.7)	-5.2, 1.6	-1.04	62.0	.304
Anthropometry									
BMI	30.0 (1.4)	29.9 (1.4)	29.0 (1.4)	29.1 (1.4)	0.15 (0.27)	-0.4, 0.7	0.56	47.0	.579
Physical activity (IPAQ scores)	2307.7 (664.8)	2251.3 (645.9)	2509.4 (636.3)	2892.2 (636.3)	439.3 (1097.6)	-1757.2, 2635	0.40	58.9	.690
Diet									
Mod*i*MedDiet (0-120)	36.2 (2.5)	55.1 (2.8)	47.3 (2.6)	45.4 (2.9)	-20.7 (4.3)	-20.7, -12.1	-4.78	55.62	<.001
Biomarkers									
Glucose (mmol/L)	4.5 (0.5)	4.7 (5.5)	5.4 (0.5)	5.5 (0.6)	-0.1 (4.3)	-0.9, 0.8	-0.10	48.5	.919
Cholesterol (mmol/L)	4.9 (0.2)	4.8 (0.2)	5.5 (0.2)	5.2 (0.2)	-0.2 (0.2)	-0.5, 0.2	-1.08	47.7	.287
Triglycerides (mmol/L)	1.3 (0.2)	1.2 (0.2)	1.4 (0.2)	1.5 (0.2)	0.2 (0.2)	-0.3, 0.7	0.88	53.4	.386
HDL cholesterol (mmol/L)	-1.5 (0.1)	1.5 (0.1)	1.4 (0.1)	1.3 (0.1)	-0.1 (0.1)	-0.2, 0.1	-1.11	47.7	.272
LDL cholesterol (mmol/L)	2.9 (0.2)	2.8 (0.1)	3.4 (0.2)	3.2 (0.2)	-0.1 (0.2)	-0.4, 0.3	-0.48	47.1	.636
Total saturates (% FA)	35.7 (0.5)	36.6 (0.5)	35.6 (0.5)	38.1 (0.6)	1.6 (1.0)	-0.5, 3.6	1.54	62.1	.128
Total monounsaturates (% FA)	23.2 (0.4)	22.2 (0.5)	22.8 (0.4)	23.2 (0.6)	1.4 (0.8)	-0.3, 3.1	1.69	55.4	.096
Total polyunsaturates (% FA)	41.1 (0.7)	41.3 (0.8)	41.6 (0.7)	38.6 (0.9)	-3.1 (1.3)	-5.7, 0.5	-2.41	54.9	.019
n6 (% PUFA)	14.6 (0.4)	14.5 (0.4)	14.3 (0.4)	12.8 (0.5)	-1.4 (0.8)	-3.0, 0.3	-1.69	62.1	.096
n3 (% PUFA)	6.9 (0.3)	6.8 (0.3)	6.5 (0.3)	7.2 (0.4)	0.9 (0.6)	-0.3, 2.1	1.57	57.8	.123

*MADRS* Montgomery-Åsberg Depression Rating Scale, *HADS* Hospital Anxiety and Depression Scale, *POMS* Profile of Mood States, *GSE* Generalized Self-Efficacy scale, *WHO-5* WHO (Five) wellbeing index, *BMI* body mass index, *FA* fatty acids, *PUFA* polyunsaturated fatty acids

^a^Derived from planned comparison within mixed model repeated measures (MMRM) using all available data (baseline data *n* = 67, 12 week data *n* = 56) and based on unadjusted estimates

On the CGI-I at 12 weeks, the dietary support group had significantly lower average scores (*M* = 2.1, *SD* = 1.3) than the social support control group (*M* = 3.0, *SD* = 1.3), *t*(50) = –2.58, *p* = 0.013. Based on these figures, the dietary support group on average had ’much improved’ scores, whereas the social support control group had ’minimally improved’ scores on the CGI-I.

On the POMS total mood disturbance score, as well as the subscale scores (subscales not reported) there were no significant differences between the groups. Similarly, there were no significant differences between groups with respect to self-efficacy or wellbeing.

At intervention cessation, the dietary support group had significant improvements in the consumption of the following food groups: whole grain cereals (mean increase 1.21 (SD 1.77) servings/day); fruit (0.46 (0.71) servings/day); dairy (0.52 (0.72) servings/day); olive oil (0.42 (0.49) servings/day); pulses (1.40 (2.39) servings/week); and fish (1.12 (2.65) servings/week). With respect to the consumption of unhealthful food items, intake of extras substantially declined (mean decrease 21.76 (SD 16.01) servings/week) in the dietary support group. Conversely, there were no significant changes observed in the social support control group for any of the key food groups. These findings were confirmed by analysis of the Mod*i*MedDiet scores: the dietary support group showed significantly greater improvement from baseline to 12 weeks on Mod*i*MedDiet scores than controls, *t*(55.6) = –4.78, *p* < 0.001; the differences remained after controlling for sex, education, physical activity, baseline BMI and baseline Mod*i*MedDiet score. Cohen’s *d* for the Mod*i*MedDiet was 1.36 (95% CI 0.74–1.98). There were no significant differences between groups with respect to BMI or physical activity.

Data on change in psychopharmacological medications over the 12 weeks were available for 53 individuals. One person in each of the dietary support and social support groups started taking psychopharmacological medications over the 12 weeks. There were two patients in the social support group who ceased their medications. There were too few participants to undertake inferential statistics. Changes in biomarkers are also detailed in Table 2. The only significant difference between the two groups was with respect to change in total polyunsaturated fatty acids; the social support group showed a significant drop in polyunsaturates over the 12 weeks, *t*(54.9) = –2.41, *p* = 0.019. Changes in MADRS did not correlate with any of the changes in biomarkers; all correlations were less than 0.2 and were not significant at the *p* < .050 level. Finally, change in dietary quality, measured using 12 week Mod*i*MedDiet score differences from baseline scores, was associated with change in depression scores in the intervention group: the interaction between group allocation and change in Mod*i*MedDiet scores after adjusting for baseline MADRS scores was statistically significant, *F*(2) = 9.6, *p* < 0.001. The correlation was only significant in the intervention group (*p* < 0.001); the unstandardised beta coefficient was –0.22 (95% CI –0.32, –0.12), indicating a 2.2 score improvement in MADRS with every 10% increase in dietary adherence.

## Discussion

These results provide preliminary RCT evidence for dietary improvement as an efficacious treatment strategy for treating major depressive episodes. We report significant reductions in depression symptoms as a result of this intervention, with an overall effect size of –1.16. These effects appear to be independent of any changes in BMI, self-efficacy, smoking rates and/or physical activity. Concordant with our primary outcome, significant improvements were also observed on self-reported depressive and anxiety symptoms and on the Clinical Global Impressions Improvement scale. Whilst other mood (POMS) and wellbeing (WHO-5) scores did not differ between groups, changes were in the expected direction and were likely affected by lack of statistical power. Critically, substantial improvements on the Mod*i*MedDiet score were evident in the dietary support group but not in the social support control group, and these changes correlated with changes in MADRS scores.

The results of this trial suggest that improving one’s diet according to current recommendations targeting depression [31] may be a useful and accessible strategy for addressing depression in both the general population and in clinical settings. Whilst there are many data to suggest that eating a more healthful diet is more expensive than a less healthful diet [45], our detailed modelling of the costs of 20 of the SMILES participants’ baseline diets compared to the costs of the diet we advocated showed that our strategy can be affordable [46]. Indeed, we estimated that participants spent an average of AU$138 per week on food and beverages for personal consumption at baseline, whilst the costs per person per week for the diet we recommended was AU$112 per week, with both estimations based on mid-range product costs [46].

A pertinent observation was that improvements in depressive symptoms were independent of weight change. These findings were expected, as the diet intervention was *ad libitum* and did not have a weight loss focus, but provide further support for the beneficial role of dietary improvement per se. The extensive observational evidence linking diet quality to mental health has repeatedly shown that the observed relationships exist independently of various measures of body composition.

Although dietary changes were not reflected in the traditional cardiovascular disease biomarkers, the protective effects of healthful dietary patterns are often independent of these risk factors [47]. There are many other biological pathways by which dietary improvement may influence depressive illness; previous discussions have centered on inflammatory [18] and oxidative stress [19] pathways, as well as brain plasticity [16] and the new evidence base focused on the gut microbiota [17]. Each of these pathways is suggested to play a role in depression and is also influenced by diet quality. Moreover, behavioural changes associated with food (cooking/shopping/meal patterns) are an expected outcome of a nutrition intervention, and these changes in activity may also have had a therapeutic benefit.

### Strengths and limitations

There are methodological features of our study that must be considered. Firstly, there is the issue of expectation bias due to the fact that we needed to be explicit in our advertising regarding the nature of the intervention and to the inability to blind the participants to their intervention group; this may have biased the results and also resulted in differential dropout rates. Moreover, in regard to our randomisation process, a block size of four, whilst recommended for small sample sizes to avoid imbalances in allocation, may have been insufficient to support allocation concealment. As discussed above, to mitigate these issues significant effort was made to mask our hypothesis from the participants, and emphasis was placed on the potential benefit of social support to mental health. Clearly, our results must also be considered in light of the small sample size. Failure to reach our planned sample size increases the possibility that our sample was not representative and limited our ability to conduct subgroup analyses. It may also have inflated the effect size we observed. However, our original power calculations were based on a very small effect size; arguably, this would not have been clinically significant. There were differential completion rates in each group: 94% versus 73.5% in the dietary and social support groups, respectively. This suggests that the mechanisms underpinning missingness may be different between the two groups; however, results from comprehensive sensitivity analyses testing alternatives to the MAR assumption revealed that, whilst under the NMAR assumptions observed intervention effects moved towards the null, our findings remained robust against departures from the MAR assumption. A larger sample size and assessments at more than two time points would have afforded more sophisticated statistical modelling; this should be a key focus of future replication studies.

Importantly, the high completion rates in the intervention group point to the acceptability of the dietary intervention to the participants. The fact that the dietary intervention group was able to make significant improvements to their diet quality suggests that dietary improvement is achievable for those with clinical depression despite the fatigue and lack of motivation that are prominent symptoms of this disorder. On the other hand, the challenges we had with recruiting this clinical population, likely due to the aforementioned symptoms and the requirement to attend the study centre on several occasions, points to the need to utilise different methods for delivering the intervention that do not require attendance with the dietician in person, such as telephone or Skype. Finally, given that we recruited participants on the basis of existing ‘poor’ quality diet, this may limit the generalisability of our findings to the wider population of individuals with depression. However, evidence suggests that our study sample was not necessarily a special subgroup; the recent 2014–2015 Australian Health Survey tells us that only 5.6% of Australian adults had an adequate intake of vegetables and fruits. In this study, only 15 out of 166 people screened were excluded on the basis of a pre-existing ‘good’ diet, suggesting that — concordant with the wider population — poor diet is the norm in those with depressive illness.

### Implications

Recent updates to clinical guidelines for the treatment of mood disorders in Australia have, in recognition of the emerging and established data regarding the importance of health behaviours (diet, exercise, sleep and smoking) to mood disorders, made explicit recommendations regarding the need to address these behaviours as a first step in the treatment of patients [48]. The results of this RCT offer further support for the need to focus on addressing poor diet in clinical practice and provide some guidance regarding the strategies that may be used to support this imperative. They suggest the new possibility of adding clinical dieticians to multidisciplinary mental health teams and making dietician support available to those experiencing depressive symptoms in primary and other care settings. Clearly, successfully improving diet quality in patients will also benefit the physical illnesses that are so commonly comorbid with depression and which are both a cause and consequence of depression. Upskilling dieticians to best deliver this program to this patient population may also be required.

## Conclusions

In summary, this is the first RCT to explicitly seek to answer the question: If I improve my diet, will my mental health improve? Whilst emphasising the preliminary nature of this study and the imperative for replication in studies with larger sample sizes, the results of our study suggest that dietary improvement guided by a clinical dietician may provide an efficacious treatment strategy for the management of this highly prevalent mental disorder. Future work in this new field of nutritional psychiatry research should focus on replication, ensuring larger samples and more sophisticated study designs, in order to confirm effects and afford sensitivity analyses to identify predictors of treatment response. The scaling up of interventions and identification of the pathways that mediate the impact of dietary improvement on depressive illness are also key imperatives. Clinicians should also consider promoting the benefits of dietary improvement and facilitating access to dietetics support for their patients with depression.
