# Correction to: A randomised controlled trial of dietary improvement for adults with major depression (the ‘SMILES’ trial)

**DOI:** 10.1186/s12916-018-1220-6

**Published:** 2018-12-28

**Authors:** Felice N. Jacka, Adrienne O’Neil, Rachelle Opie, Catherine Itsiopoulos, Sue Cotton, Mohammedreza Mohebbi, David Castle, Sarah Dash, Cathrine Mihalopoulos, Mary Lou Chatterton, Laima Brazionis, Olivia M. Dean, Allison M. Hodge, Michael Berk

**Affiliations:** 1 IMPACT Strategic Research Centre, Deakin University, Geelong, VIC Australia; 2 School of Population Health, The University of Melbourne, Melbourne, VIC Australia; 3 Orygen, The National Centre of Excellence in Youth Mental Health, Parkville, VIC Australia; 4 Department of Psychiatry, University of Melbourne, Melbourne, VIC Australia; 5 School of Allied Health, La Trobe University, Melbourne, VIC Australia; 6 Department of Medicine, The University of Melbourne, Melbourne, VIC Australia; 7 Centre for Population Health Research, Deakin University, Geelong, VIC Australia; 8 Cancer Epidemiology and Intelligence Division, Cancer Council Victoria, Carlton, VIC Australia; 9 Centre for Adolescent Health, Murdoch Childrens Research Institute, Melbourne, VIC Australia; 10 Black Dog Institute, Randwick, NSW Australia; 11 St Vincents Hospital, Fitzroy, VIC Australia; 12 The Florey Institute of Neuroscience and Mental Health, Parkville, VIC Australia; 13 Food & Mood Centre, Deakin University, IMPACT SRC, School of Medicine, PO Box 281, Geelong, VIC 3220 Australia

## Correction

The original version of this paper [1] did not specify that a website was used in the final year of recruitment, in addition to the other stated recruitment methods. The current version of this website has the title ‘Healthy eating as a new approach to treating depression’ and can be found here: https://dietdepressionstudy.com/. Twelve participants were recruited after the establishment of this website (June 2014), of whom one reported the website as their recruitment point. In addition to the three media interviews listed on the recruitment website, a fourth interview, which also provided details for recruitment, can be found here: https://www.pressreader.com/australia/geelong-advertiser/20121018/textview

The authors also wish to clarify additional points about their paper. With regards to the information that participants were given about the study hypothesis after recruitment, the Participant Information and Consent form (PICF) stated: “Research has shown that poor diet is closely linked to poor mental health. Improving the quality of a person’s diet may lead to better mental health including reduced depression. However, this has not yet been established”. Regarding the information that was withheld from the study participants relating to the study hypotheses, this was that no information about content of the diet was provided prior to the study to either group. The potential benefits of social support to mental health were not presented during recruitment, but were discussed with participants when they were given the Participant Information and Consent form (PICF).
